# Rapid and simultaneous detection of multiple pathogens in the lower reproductive tract during pregnancy based on loop-mediated isothermal amplification-microfluidic chip

**DOI:** 10.1186/s12866-022-02657-0

**Published:** 2022-10-29

**Authors:** Xiaofang Xu, Yiguo Jia, Ruolin Li, Yuting Wen, Yuchen Liang, Guangjie Lao, Xiaojuan Liu, Wei Zhou, Huawei Liu, Jiang Xie, Xiaoxia Wang, Wenming Xu, Qun Sun

**Affiliations:** 1 Key Laboratory of Bio-Resources and Eco-Environment of the Ministry of Education, College of Life Sciences, Sichuan University, Chengdu, 610065 Sichuan China; 2 Department of Obstetrics/Gynecology, Joint Laboratory of Reproductive Medicine, SCU-CUHK, Key Laboratory of Obstetric, Gynaecologic and Paediatric Diseases and Birth Defects of Ministry of Education, West China Second University Hospital, Sichuan University, Chengdu, 610041 China; 3 School of Life Sciences and Biotechnology, Shanghai Jiao Tong University, Shanghai, China; 4 Department of Laboratory Medicine, West China Second University Hospital, Sichuan University, Chengdu, China; 5 Key Laboratory of Birth Defects and Related Diseases of Women and Children, Sichuan University, Ministry of Education, Chengdu, China; 6 The Third People’s Hospital of Chengdu, Affiliated Hospital of Southwest Jiao Tong University, Chengdu, China; 7 Tianjin Chase Sun Pharmaceutical Co., Ltd., No. 20 Quanfa Road, Tianjin, 301700 China

**Keywords:** Reproductive tract infection, Pathogen, Loop-mediated isothermal amplification, Microfluidic technology, LAMP-microfluidic chip, Rapid and simultaneous detection

## Abstract

**Background:**

Female reproductive tract infection (RTI) is the common source of varied diseases, especially as an important risk factor for pregnancy outcomes, therefore the rapid, accurate and simultaneous detection of multiple pathogens is in urgent need for assisting the diagnosis and treatment of RTI in pregnant women. *Streptococcus agalactiae* (*S*. *agalactiae*), *Enterococcus faecalis* (*E*. *faecalis*), *Gardnerella vaginalis* (*G*. *vaginalis*), *Candida albicans* (*C*. *albicans*) and *Chlamydia trachomatis* (*C*. *trachomatis*) are five main pathogens in lower genital tract with high risk, serious consequences and clinical demands. The combination of loop-mediated isothermal amplification (LAMP) and microfluidic technology was used to develop the LAMP-microfluidic chip for rapid, simple, sensitive and simultaneous detection of the five target pathogens above.

**Results:**

Standard strains and clinical isolates were used for the establishment of the novel LAMP method in tube and LAMP-microfluidic chip, followed by the chip detection on 103 clinical samples and PCR verification partially. The sensitivities of LAMP of *S*. *agalactiae*, *E*. *faecalis*, *G*. *vaginalis*, and *C*. *albicans* in tube were 22.0, 76.0, 13.2, 1.11 CFU/μL, respectively, and *C*. *trachomatis* was 41.3 copies/μL; on LAMP-microfluidic chip they were 260, 154, 3.9 and 7.53 CFU/μL, respectively, and *C*. *trachomatis* was 120 copies/μL. The positive coincidence rates of clinical stains in tube and on chip experiments were 100%. Compared with the classic culture method performed in hospitals, the positive coincidence rate of the 103 clinical samples detected by LAMP-microfluidic chip were 100%. For the six inconsistent ones, including four *G*. *vaginalis* and two *C*. *albicans* positive samples tested by LAMP-microfluidic chip and verified by PCR were negative by culturing method in hospitals, indicating the lack of efficient detection by the classic culturing method.

**Conclusion:**

Our study suggested that the LAMP-microfluidic chips could simultaneously, efficiently, and accurately detect multiple main pathogens, including *S*. *agalactiae*, *E*. *faecalis*, *G*. *vaginalis*, *C*. *albicans* and *C*. *trachomatis*, in clinical samples of female RTI to give a great clinical value. Accordingly, this novel method has the potential to provide a valuable reference for female RTI screening and early diagnosis during pregnancy.

**Supplementary Information:**

The online version contains supplementary material available at 10.1186/s12866-022-02657-0.

## Background

A series of infectious diseases that occur in the reproductive system are collectively called reproductive tract infection (RTI). As a global social and public health issue, female RTI is the general term for female reproductive system infected by pathogens such as bacteria, virus, and *Candida*, which is the source of many diseases and is mainly characterized by high incidence, high recurrence rate and wide epidemic range [1]. A number of studies have confirmed that microbial infections including *Streptococcus agalactiae* (*S*. *agalactiae*, Group B *Streptococcus*, GBS), *Enterococcus faecalis* (*E*. *faecalis*), *Gardnerella vaginalis* (*G*. *vaginalis*), *Candida albicans* (*C*. *albicans*) and *Chlamydia trachomatis* (*C*. *trachomatis*) during pregnancy are important risk factors for pregnancy outcomes, such as miscarriage, premature birth, newborn infant infections and other complications, which can seriously affect the health of both the mother and fetus [2–4]. *S*. *agalactiae* is an important cause of sepsis during the puerperium of pregnant women and neonatal meningitis [5]. In 2002, USA Centers for Disease Control and Prevention recommended universal screening for GBS colonization at 35–37 weeks of gestation [6]. Among the more than 3,000 strains of pathogens isolated by Peking Union Medical College Hospital from bacterial infections in the female reproductive tract from 1990 to 2009, the detection rate of *E*. *faecalis* was 14%, ranking third [7]. In 2017, *G*. *vaginalis* and *C*. *albicans*, two of the main pathogens, were isolated from both the obstetric ward and the genital secretions of pregnant women of the Second Hospital of West China Affiliated Hospital of Sichuan University. *G*. *vaginalis*, as the most important pathogens of bacterial vaginitis, damages female reproductive health and is closely related to premature birth and other adverse pregnancy outcomes [8, 9]. *C*. *albicans* increases its adaption and invasion ability to cause disease when there is impaired immunity or the normal flora is imbalanced [10]. *C*. *albicans* infection in pregnant women can go up through the cervix and penetrate the fetal membrane to cause premature, and even cause oral infection in newborns after passing through the vagina [11, 12]. *C*. *trachomatis* infection during pregnancy is almost asymptomatic, but can cause premature delivery, premature rupture of membranes, low birth weight or stillbirth, and further cause neonatal pneumonia and inclusion body conjunctivitis [13, 14]. Screening for *C*. *trachomatis* has been recommended by both the American Academy of Obstetrics and Gynecology and the American Academy of Maternal and Fetal Sciences in the first three months of pregnancy with regarding to its high infection rate in pregnant women of up to 35% [15]. Single or co-infection by these pathogens mentioned above may affect the health of the mother and fetus, so making a specifically-targeted detection is necessary.

So far, the detection and identification of pathogens still largely rely on culturing in vitro, the Koch's law, which is mature enough and the gold standard for microbial detecting [16]. However, microbial culture requires complete equipment, complicated and professional operations, and long detection time. In cases needing immediate results, such as clinical screening and food industry applications, detection based on culture methods cannot yet meet the demands [17]. The culture method is also difficult for the detection of certain difficult or non-cultivable pathogens, such as *C. trachomatis*. The rapid development of molecular biology detection methods has been widely used [18]. At present, many pathogen detection methods based on Polymerase Chain Reaction (PCR) or PCR-derived technology have also become the golden standard [19–21]. However, compared with PCR and its derivative technologies, which need instruments and equipments with high requirements for operators, loop-mediated isothermal amplification (LAMP) with constant temperature amplification is simple, fast, economical but accurate and specific. Four primers (F3, B3, FIP = F1c + F2, BIP = B1c + B2) can identify six different regions, and if one or both of the loop primers (LF and LB) are added, the efficiency of target gene amplification can be significantly improved [22]. The LAMP reaction can achieve 10^9^ ~ 10^10^ times sequence amplification in a reaction time ranging from 15 to 60 min [23]. By adding commonly used dyes, SYBR Green I or EvaGreen [24], real-time visualization of the LAMP reaction can be realized. In terms of clinical diagnosis, the World Health Organization (WHO) expert group evaluated the efficiency, sensitivity and accuracy of LAMP detection in *Mycobacterium tuberculosis* in 2013, and recommended it as an immediate detection method to replace the currently used sputum smear microscopy to diagnose tuberculosis [25].

Microfluidic technology integrates the sample preparation, reaction, separation and detection together by controlling the fluid movement in the microfluidic chip. Microfluidic devices have several advantages over conventional macro-scale devices, the small size of the microchannel enables liquid flow to be dominated by a stable laminar flow, requires smaller amount of samples and controls heat and mass transfer well [26–28]. Based on these advantages, the combination and integration of LAMP detection of multiple pathogens and microfluidic chips can provide a good application prospect for nucleic acid detection and analysis [29]. In recent years, the application of LAMP-microfluidic chips has mainly focused on the detection of the aquaculture and food-borne pathogens [30–33], including *Vibrio*, *Aeromonas hydrophila*, *Edwardsiella tarda*, *Listeria monocytogenes*, *Escherichia coli* (*E*. *coli* O157:H7), *Salmonella* and *Staphylococcus aureus* (*S*. *aureus*), but has been rarely applied to the female RTI during pregnancy, where there is an urgent need for early rapid screening technology to decrease the risk.

This research aims to develop a rapid detection method for the pathogens of the lower genital tract during pregnancy. Five species of pathogens in the lower genital tract with high risk, serious consequences and realistic demand were selected as the detection targets, including *S*. *agalactiae*, *E*. *faecalis*, *G*. *vaginalis*, *C*. *albicans* and *C*. *trachomatis*. By utilizing the rapid, sensitive and simple characteristics of LAMP method, the detection method of five species of pathogens has been developed. Through the combination of LAMP and microfluidic technology, it is expected that five pathogens can be efficiently, highly sensitively and simultaneously detected, and an accurate, rapid and simple detection method is provided for female RTI.

## Results

### Screening and determination of the pathogens

For women at a certain stage of pregnancy, different pathogenic microbial infections have corresponding effects on the mother and fetus [34]. After comprehensively considering the risk of RTI during pregnancy and the opinions of clinicians, five high-risk detection target pathogens with serious consequences and clinical application demands, including *S*. *agalactiae*, *E*. *faecalis*, *G*. *vaginalis*, *C*. *albicans* and *C*. *trachomatis*, were finally determined.

### Determination of the target genes and primers for LAMP detection

The glucokinase gene (*glk*) is one of the housekeeping genes of *S*. *agalactiae*. Studies have determined the variable nucleotide positions of the *glk* gene of 152 *S*. *agalactiae* strains, whose percentage is 1.5%. The *glk* gene is ubiquitous and conserved in *S*. *agalactiae* [35], so that it can be used as a specific gene for the identification of *S*. *agalactiae*. The *gyrA* gene encoding the A subunit of the DNA gyrase is a relatively conservative housekeeping gene and the expression stability of *gyrA* is second only to 16S rRNA [36]. The phylogenetic analysis and strain identification of *Bacillus* usually rely on the *gyrA* gene [37], making it possible to be used as a specific gene for the identification of *E*. *faecalis* and *C*. *trachomatis*. The sequence similarity of the *gyrA* gene of the two serovar types of *C*. *trachomatis* serotype D and L2 (Gene ID: 884,941; Gene ID: 5,859,599 and Gene ID: 5,858,742) published in the NCBI-Gene database is 99.56%, which is very high and quite different from other *Chlamydia* (Table 1), so that it can be used for the identification of *C*. *trachomatis*. Research [38] has proven that the LAMP method based on the 23S rRNA gene sequence can specifically detect *G*. *vaginalis*, which offered a novel method for rapid diagnosis of *G*. *vaginalis* not only in the laboratory but also in outpatient clinical conditions. A study [39] selected LAMP primers designed to target the internal transcribed spacer 2 (ITS2) region between the 5.8S and 26S rRNA genes, in which the constructed LAMP method can detect *Candida albicans* in contaminated dairy products within 1 h, and will not have non-specific reactions with other *Candida*. After the primers of the *glk* gene of *S*. *agalactiae*, and the *gyrA* gene of both *E*. *faecalis* and *C*. *trachomatis* were designed, DNAMAN software was used to compare the similarity between the corresponding target gene of both the target pathogens and their relative microorganisms. The comparison results showed that the gene similarity was about 62% ~ 83% (Table 1). From the comparison of the genes in these closely related microorganisms, the selected gene sequence showed dramatical differences among species, and this partially contributed to the specificity of the LAMP primers designed.

**Table 1:** Similarity comparison for *Streptococcus agalactiae*, *Enterococcus faecalis*, and *Chlamydia trachomatis*

Target pathogen	Related microorganisms of the same genus	Similarity of target gene (%)
*S. agalactiae*	*Streptococcus pharyngitis*	63.57
	*Streptococcus pneumoniae*	62.01
	*Streptococcus pyogenes*	75.51
*E. faecalis*	*Enterococcus faecium*	75.96
	*Enterococcus durable*	76.40
	*Enterococcus avium*	75.65
*C. trachomatis*	*Chlamydia pneumoniae*	72.61
	*Chlamydia psittaci*	74.52
	*Chlamydia suis*	83.72

The primers of *glk*-*4* designed for the glucokinase gene of *S. agalactiae*, the *gyrA-3* for the gyrase subunit A gene of *E. faecalis*, and the *gyrA*-*5* for the gyrase subunit A gene of *C. trachomatis*, were screened out (Table 2). But the 23S-r primer of *G*. *vaginalis* and the ITS primer of *C. albicans* were from references (Table 2). The primer BLAST was used to verify the specificity of the primers. F2-B2 and F3-B3 pairs of the *glk*-*4* in GBS only matched *S*. *agalactiae*. F3-B3 pair of the *gyrA*-*3* in *E*. *faecalis* only matched *E*. *faecalis*, but *Lactobacillus herbarum* had four bases apart to match. F2-B2 pair of *E*. *faecalis* can additionally match *Enterococcus phoeniculicola*. However, the two species above generally do not exist in the human body, neither the hospital environment, thus they have very limited impact on the detection. F2-B2 and F3-B3 pairs of the *gyrA-5* in *C. trachomatis* only matched with that of *C*. *trachomatis*. The blasting results of the LAMP primers designed for *S. agalactiae*, *E. faecalis* and *C. trachomatis* proved the good specificity of the primers, but they should continue to be evaluated in following experiments.

**Table 2:** LAMP primers of five target pathogens

Pathogen	Primer	Sequence
*S. agalactiae*	F3-*glk***-**4	AACATCGTTTGAGCCTCTA
	B3-*glk-*4	ATTATTGGCACCAGCACC
	FIP-*glk-*4	AGTTCTATCAACAGCTCCTGGAGATTAACAAAAGATGACTTTCTCG
	BIP-*glk*-4	GGCTGATACTCAAGAAGTAGGTTCCTGCAACATTAGCATCGTTAT
*E. faecalis*	F3-*gyr*A-3	GCAATCAGAAGATGATTATCGG
	B3-*gyr*A-3	GCTTACCATAACCATTTTCAGT
	FIP-*gyr*A-3	GATGCTGTCCGGCCCATATCCACATGCAGGATACTCTGTC
	BIP-*gyr*A-3	GGAATCCGTCTCCGCGAAAAACTAGGACTTCTTTATTTTCATCCA
*G. vaginalis*	F3-*23S*-r	CGGGTTGATATTCCCGTACC
	B3-*23S*-r	ACATCCCCCGGATTTACCT
	FIP-*23S*-r	AACCCCGAAAGGTAATCAACCAGGGTGTTACACCGTTCGAACTG
	BIP-*23S*-r	TCGGTAGTAGGTTAGCGTGGGAGGACAGCCCACACGCTTAG
	LF-*23S*-r	ACGAAGGTTAGTACTCTCAGATT
	LB-*23S*-r	CGGCTGCGGAGGTGGTTTT
*C. albicans*	F3-ITS	TCTGGTATTCCGGAGGGC
	B3-ITS	AGTCCTACCTGATTTGAGGT
	FIP-ITS	CTACCGTCTTTCAAGCAAACCCATGAGCGTCGTTTCTCCCT
	BIP-ITS	TTGACAATGGCTTAGGTCTAACCAAAAGATATACGTGGTGGACGTTAC
	LB-ITS	CTCAACACCAAACCCAGCGG
*C. trachomatis*	F3-*gyr*A-5	TTACTCCGCATCCACGAA
	B3-*gyr*A-5	AACACCCTTCTTGGTAGC
	FIP-*gyr*A-5	ACGTTCTCCTTCAGGAAGCTGGATCTTCACCAACTTCGGT
	BIP-*gyr*A-5	TCCTGGAAGGAATCCGTCCCGGTATTCTCCTTGCTCGAAC
	LB1-*gyr*A-5	GAGAACAAGTTGCTGCGGTGCT

### LAMP in tube

After the culture and colony count, the concentrations of *S*. *agalactiae*, *E*. *faecalis*, *G*. *vaginalis* and *C*. *albicans* were 2.20 × 10^9^, 7.60 × 10^8^, 1.32 × 10^9^, and 1.11 × 10^7^ CFU/mL. For the *C. trachomatis*, the concentration of PCR product after Gel extraction was 59.53 μg/mL, which was equivalent to 4.13 × 10^10^ copies/μL. All the strains above after DNA extraction and the quantitative target fragments of *C*. *trachomatis* were used for the tube experiments except for the repeatability tests.

### Specificity

**Fig. 1:**
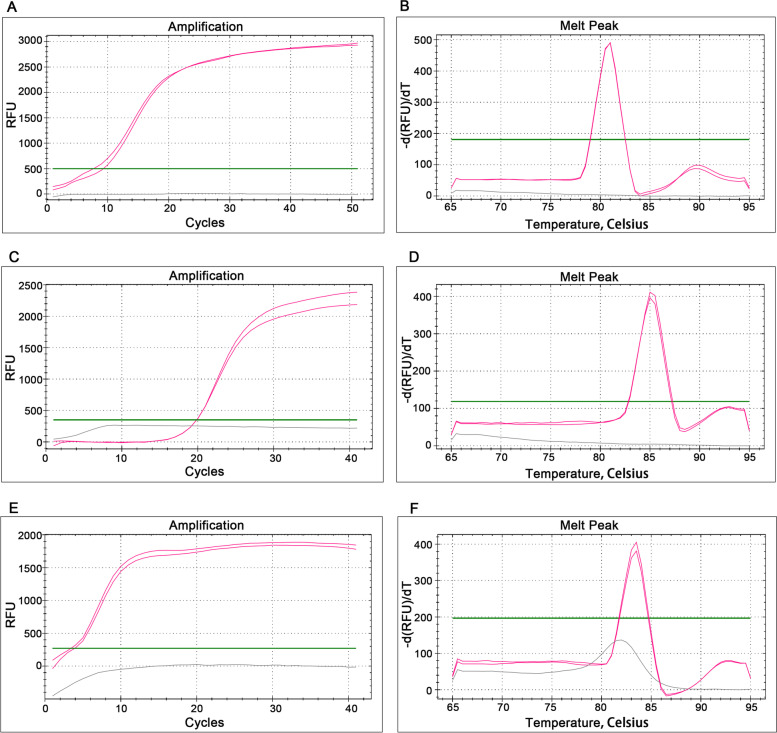
Amplification curves and melting curves of *S*. *agalactiae*, *E*. *faecalis*, and *C*. *trachomatis*. **A, C, E** Amplification curves of *S*. *agalactiae*, *E*. *faecalis*, and *C*. *trachomatis*. **B, D, F** Melting curves of *S*. *agalactiae*, *E*. *faecalis*, and *C*. *trachomatis*. The red curves represent the target pathogens and the gray curves represent the blank control

**Fig. 2:**
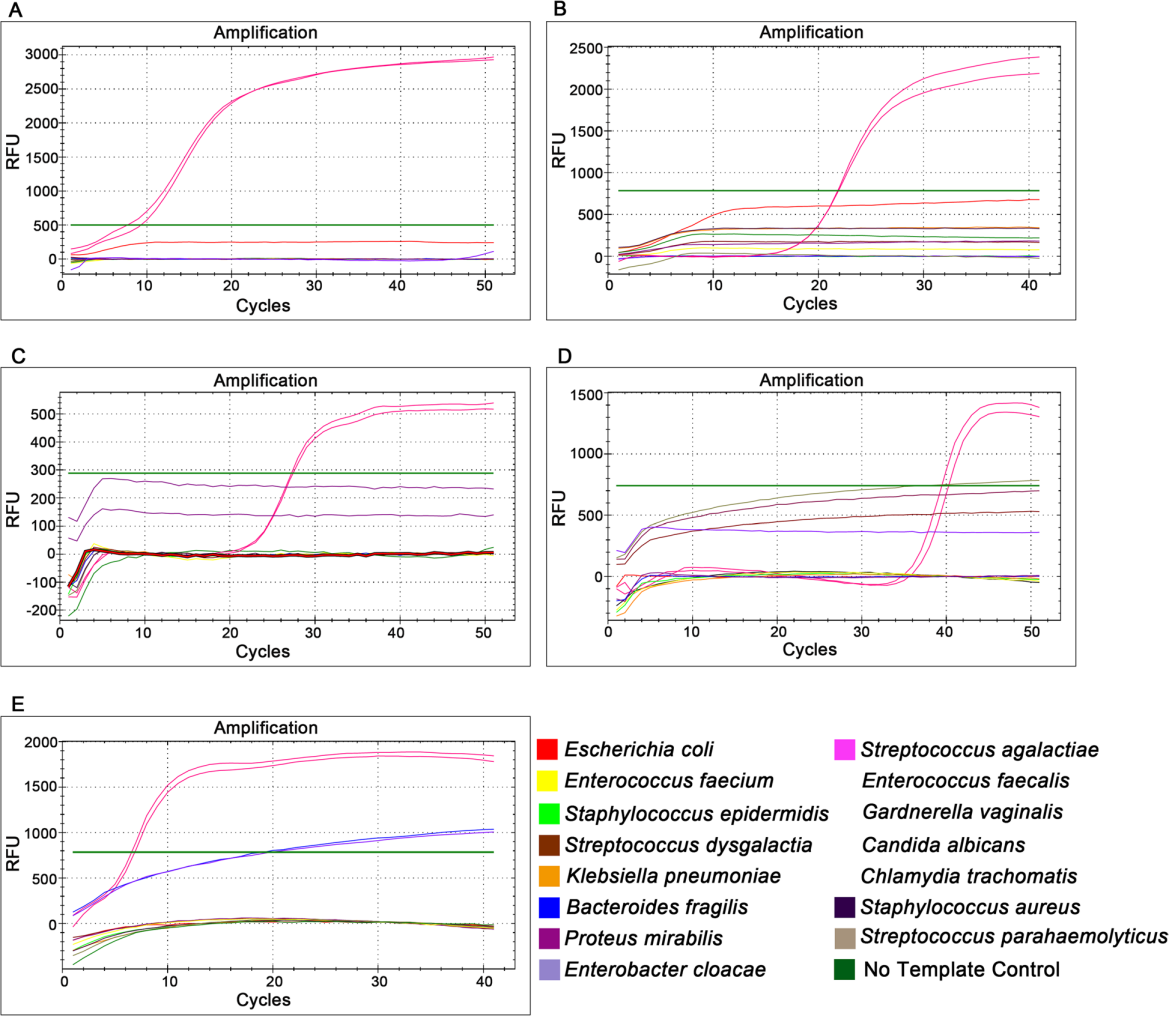
Specificity of LAMP reaction for five target pathogens. **A** *Streptococcus agalactiae*, **B** *Enterococcus faecalis*, **C** *Gardnerella vaginalis*, **D** *Candida albicans*, **E** *Chlamydia trachomatis*

For the three primer sets designed in this study, the melting curves of the LAMP positive amplification products of *S. agalactiae, E. faecalis and C. trachomatis* had only one melting peak (Fig. 1), indicating that there were no non-specific products. In addition, the melting curves of both amplification products and blank controls did not show any messy peaks at less than 80 °C, confirming the good quality of the primer sets. In particular, all the melting curves of the *C. trachomatis* blank controls after amplification had a peak (Fig. 1F), which might be caused by the production of primer dimers. Despite this, the blank control of *C*. *trachomatis* did not form amplification curve (Fig. 1E), indicating that the production of primer dimers did not affect the specificity of the *C*. *trachomatis* LAMP reaction. More importantly, the DNA of each target pathogen and ten kinds of common RTI pathogens were added into the corresponding LAMP reaction system of five target pathogens for amplification to further test the specificity of LAMP method. It could be seen from the “S” shaped amplification curves (Fig. 2) that all the targets were amplified within 40 min and no non-specific amplification occurred. LAMP curves appeared only in the reactions with the DNA of target strains, which showed that the LAMP reaction of the five detection targets had good specificity.

### Sensitivity

**Fig. 3:**
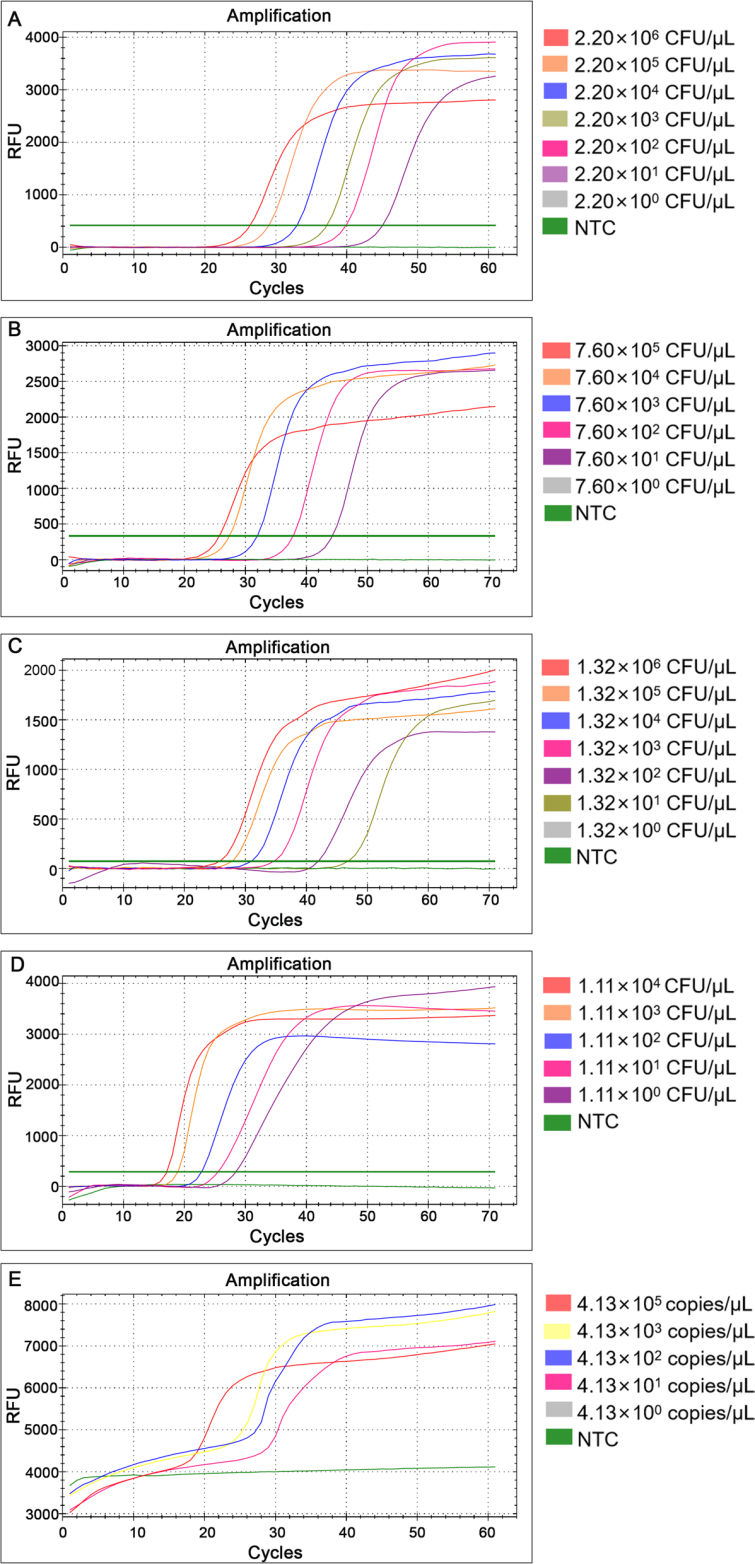
Sensitivity of LAMP detection for **A** *S*. *agalactiae*, **B** *E*. *faecalis*, **C** *G*. *vaginalis*, **D** *C*. *albicans*, **E** *C. trachomatis*

Each gradient dilution of DNA was amplified three times. From the curves (Fig. 3), it could be seen that as the template concentration decreased, the reaction time and the Cycle threshold (Ct) values gradually increased. The LAMP sensitivities of *S*. *agalactiae*, *E*. *faecalis*, *G*. *vaginalis* and *C*. *albicans* were 22.0, 76.0, 13.2 and 1.11 CFU/μL respectively. In particular, different concentrations of target fragments were used for the sensitivity test of *C*. *trachomatis*, the LAMP sensitivity was 41.3 copies/μL.

### Repeatability

The concentrations of PCR purified products of *S*. *agalactiae*, *E*. *faecalis*, *G*. *vaginalis*, *C*. *albicans* and *C*. *trachomatis* were 1.03 × 10^11^, 1.60 × 10^11^, 2.54 × 10^11^, 1.62 × 10^11^ and 3.30 × 10^9^ copies/μL. The diluted target DNA fragments were used for 20 repetitive amplifications and the coefficient of variation (CV) values of the Ct values were counted (Table 3). The CV values of *S*. *agalactiae*, *E*. *faecalis*, *G*. *vaginalis*, *C*. *albicans* and *C*. *trachomatis* were 6.10%, 8.24%, 4.74%, 9.82% and 7.23%, respectively, indicating that LAMP method was stable and repeatable.

**Table 3:** Repeatability of the LAMP in tube

No	*S*. *agalactiae*	*E*. *faecalis*	*G*. *vaginalis*	*C*. *albicans*	*C*. *trachomatis*
1	16.86	14.26	22.10	8.58	9.10
2	16.72	13.08	23.92	14.36	10.25
3	16.50	13.41	20.13	13.91	9.92
4	15.82	13.10	21.84	14.16	8.36
5	15.69	13.13	21.90	14.31	10.42
6	15.82	13.55	20.99	14.36	9.82
7	15.80	13.48	22.09	14.39	9.35
8	15.79	13.34	20.09	15.01	8.59
9	12.18	13.19	20.84	14.01	8.71
10	16.68	13.34	20.72	13.59	9.02
11	16.13	13.47	20.03	14.17	8.86
12	16.70	13.20	20.86	13.97	8.75
13	16.29	11.59	20.30	14.10	8.92
14	16.50	11.82	20.10	13.95	9.75
15	15.96	11.37	20.31	14.21	8.03
16	15.79	13.05	20.91	13.43	10.16
17	15.71	14.20	19.84	15.16	9.50
18	16.31	13.34	20.79	14.09	9.59
19	16.37	13.38	21.67	13.59	9.44
20	16.32	9.67	21.52	15.64	10.12
Mean/min	16.00	12.95	21.05	13.95	9.33
Standard deviation/min	0.98	1.07	1.00	1.37	0.67
CV/%	6.10	8.24	4.74	9.82	7.23

*CV* Coefficient of variation

### Positive coincidence rate

For positive coincidence rate measurement, 20 clinical isolates, including five clinical isolates for each of *S*. *agalactiae*, *E*. *faecalis*, *G*. *vaginalis* and *C*. *albicans*, and five *C*. *trachomatis* clinical samples were used. All the positive coincidence rates of the five detection targets were 100%, indicating that the LAMP detection was clinically universal.

### LAMP on chip

After the culture and colony count, the concentration of *S*. *agalactiae*, *E*. *faecalis*, *G*. *vaginalis* and *C*. *albicans* were 2.60 × 10^8^, 1.54 × 10^9^, 3.90 × 10^7^, and 7.53 × 10^7^ CFU/mL. For *C. trachomatis*, the concentration of PCR product after Gel extraction was 17.25 μg/mL, which was equivalent to 1.20 × 10^10^ copies/μL. All the strains above after DNA extraction and the quantitative target fragments of *C*. *albicans* were used for the chip experiments.

### Specificity of LAMP-microfluidic chip

**Fig. 4:**
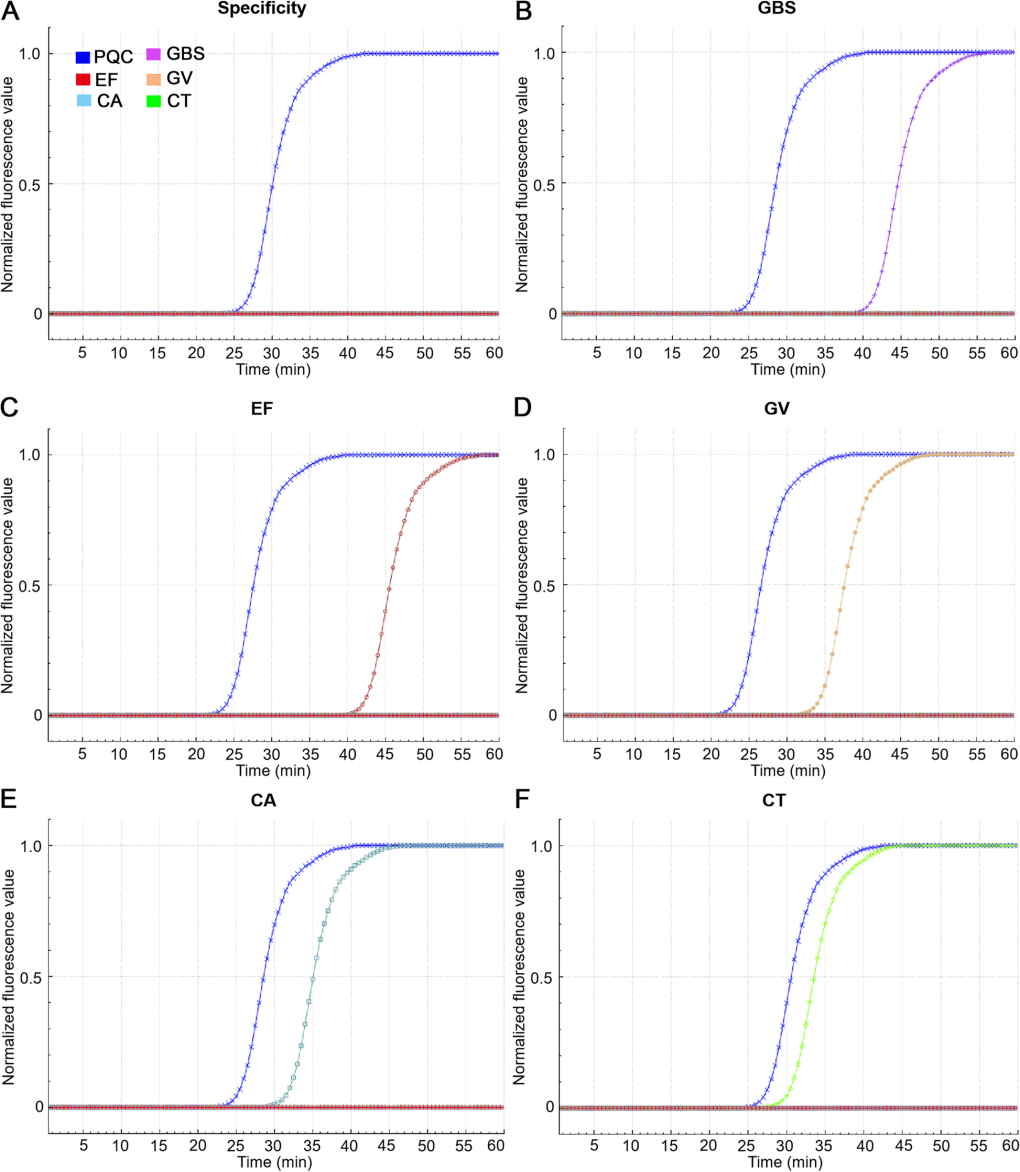
The specificity and positive amplification curves of five target pathogens on LAMP-microfluidic chip. **A** The specificity test of LAMP reaction on chip. **B ~ F** Positive amplification of GBS, EF, GV, CA and CT on chip. PQC, positive quality control. CA, *C*. *albicans*. CT, *C*. *trachomatis*. EF, *E*. *faecalis*. GBS, *S*. *agalactiae*. GV, *G*. *vaginalis*

None of the ten RTI-related pathogens had non-specific amplification on chip, and only the positive quality control was normally amplified (Fig. 4A). The positive amplifications specifically occurred on the chips in which the target DNA was added, and the amplification results were consistent with the LAMP reaction in the tube, which proved the specificity of the LAMP-microfluidic chip.

### Sensitivity of LAMP-microfluidic chip

Each dilution of DNA was repeatedly amplified three times, and the minimum concentrations that all three amplification curves occurred, were determined as the sensitivities. The positive amplification curves of five targets were shown in Fig. 4. The sensitivity results of *S*. *agalactiae*, *E*. *faecalis*, *G*. *vaginalis*, and *C*. *albicans* were 260, 154, 3.9 and 7.53 CFU/μL, and the one of *C*. *trachomatis* was 120 copies/μL.

### Repeatability of LAMP-microfluidic chip

Both the weak positive and medium positive DNA templates (three and 30 times of the sensitivity concentration) were selected as the templates of the repeatability tests. The 30 times of the sensitivities of *S*. *agalactiae*, *E*. *faecalis*, *G*. *vaginalis*, *C*. *albicans* and *C*. *trachomatis* were 7.80 × 10^3^, 4.62 × 10^3^, 1.17 × 10^2^ and 2.26 × 10^2^ CFU/μL, and 3.60 × 10^3^ copies/μL. Count the time of positive (Tp) values of ten repeated amplifications on the chip (Table 4), and calculate the CV values of five target pathogens. The CV values of the Tp values of *S*. *agalactiae*, *E*. *faecalis*, *G*. *vaginalis*, *C*. *albicans*, and *C*. *trachomatis*, were 8.91%, 4.85%, 5.63%, 3.10% and 4.82% for the medium positive templates, and 7.96%, 6.63%, 9.69%, 7.56% and 9.87% for the weak positive templates, indicating that the chips were stable and repeatable with good precision.

**Table 4:** Repeatability of the LAMP on chip

Concentration	No	PQC*	GBS	EF	GV	CA	CT
Medium positive	1	15.5	11.0	13.0	17.0	15.0	8.0
	2	16.5	10.5	12.0	16.0	15.5	8.0
	3	15.5	11.5	13.5	15.5	15.5	8.0
	4	17.5	11.5	13.0	17.0	15.0	8.0
	5	16.5	8.5	12.5	17.0	14.5	8.0
	6	17.0	11.0	12.5	17.0	14.5	9.0
	7	17.5	10.0	15.0	15.5	14.5	8.0
	8	19.5	10.0	13.0	18.5	15.0	8.0
	9	15.5	9.5	14.0	16.0	16.0	9.0
	10	16.0	10.0	13.0	16.5	14.5	9.0
	Mean /min	16.7	10.35	13.15	16.6	15.0	8.3
	SD/min	1.25	0.94	0.85	0.91	0.53	0.48
	CV/%	7.50	9.12	6.48	5.46	3.51	5.82
Weak positive	1	20.5	32	51.5	38.5	20.5	40
	2	21.5	29	53	38	23	37.5
	3	23.5	34	51.5	39	20	34
	4	22	35.5	51.5	43	22.5	29.5
	5	20.5	28.5	48	38	23	33.5
	6	22	31.5	49	39.5	20.5	31
	7	24	33.5	47	39.5	19.5	35.5
	8	21.5	29.5	45.5	48	19.5	37.5
	9	21	33	42.5	33	20.5	34.5
	10	21	35.5	48.5	39.5	18.5	30.5
	Mean/min	21.75	32.2	48.8	39.6	20.75	34.35
	SD/min	1.18	2.56	3.23	3.84	1.57	3.39
	CV/%	5.45	7.96	6.63	9.69	7.56	9.87

Medium positive, 30 times of the sensitivity. Weak positive, three times of the sensitivity. *SD* Standard deviation, *CV* Coefficient of variation, *PQC* Positive quality control, *Streptococcus agalactiae* (*GBS*), *Enterococcus faecalis* (*EF*), *Gardnerella vaginalis* (*GV*), *Candida albicans* (*CA*), *Chlamydia trachomatis* (*CT*)

### Positive coincidence rate of LAMP-microfluidic chip

For positive coincidence rate measurement on chip, five clinical isolates for each of *S*. *agalactiae*, *E*. *faecalis*, *G*. *vaginalis*, and *C*. *albicans*, and five *C*. *trachomatis* positive clinical samples were used. Each chip detected one clinical isolate or sample. All the positive coincidence rates of the five detection targets were 100%, indicating that the chip was clinically universal, as well as the previous tube reaction.

### Clinical sample test on chip and PCR verification

Among 103 clinical samples detected on LAMP-microfluidic chips, 92 were negative and 11 positive (Table 5, Additional File 1). Compared with the culture results performed in the hospital, the 95 samples detected by chip (92 negative and three positive) were consistent, but other eight positive ones were inconsistent. In other words, the negative coincidence rate was 92%, the positive was 100%, and the total was 92.23% (Table 5). Due to the restriction of the samples, only six of the eight inconsistent samples were further evaluated by clinically approved PCR method to determine that this was caused by either the clinical test failed to culture successfully or the LAMP-microfluidic chip test gave false positive results.

**Table 5:** Clinical compliance rate of LAMP-microfluidic chip detection

LAMP-Microfluidic chip	Culture in the hospital	
	+	-
+	3	8
-	0	92
In total	3	100
Negative coincidence rate: 92/100 = 92%		
Positive coincidence rate: 3/3 = 100%		
Total coincidence rate: (3 + 92)/103 = 92.23%		

**Fig. 5:**
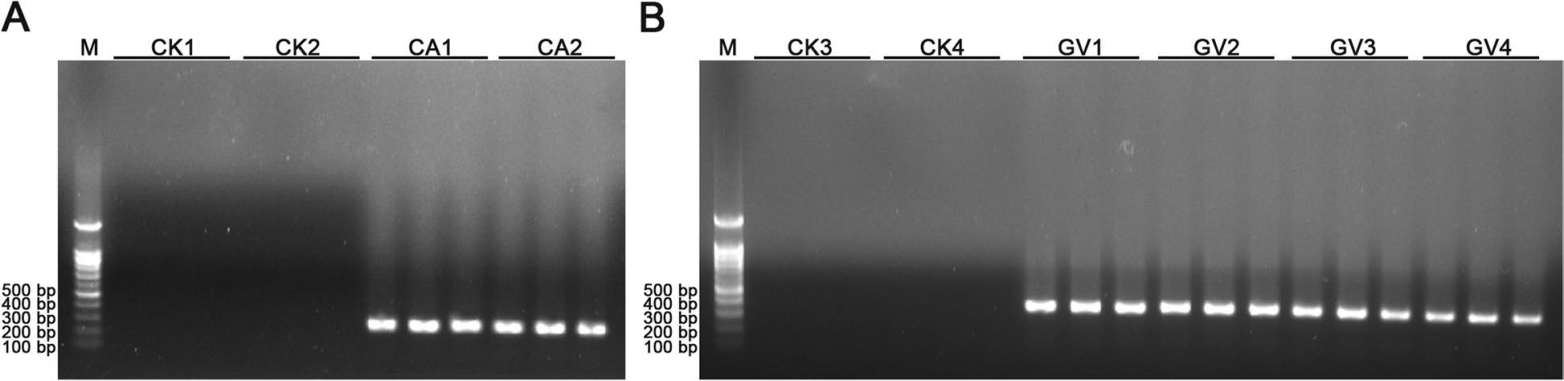
Agarose gel electrophoresis of PCR products in the validation experiments of clinical samples with positive chip results for **(A)** *C. albicans* and **(B)** *G. vaginalis*. **M**, 100 bp DNA ladder. **CK1 and CK3**, sterile water as amplification templates. **CK2 and CK4**, nucleic acid extraction reagent as amplification templates. **CA1 and CA2**, *C. albicans* PCR products of sample 1 and 80. **GV1 ~ GV4**, *G. vaginalis* PCR products of sample 5, 11, 33, 72. The gels were cropped and the original gels were presented in Additional file 2 and 3

Firstly, three clinically positive samples were randomly selected to evaluate the performance of the chip. Their mean and standard deviation of Tp values were counted, and the CV values were 5.39%, 3.67%, and 6.37%, respectively (Table 6). All the CV values of the amplifications were lower than 10%, indicating that the measurement on chips were stable for the detection of clinically positive samples. Then, the clinical samples with chip results inconsistent with the hospital test ones were verified by PCR. These non-conforming samples included six samples negative for *G*. *vaginalis* and two for *C*. *albicans* identified by the hospital culture but positive by chip detections (Table 5, Additional File 1). Among the four *G*. *vaginalis* samples, all were verified by PCR as positive (GV1 ~ GV4 in Fig. 5B, the 23S rRNA gene amplified fragment of *G*. *vaginalis* was 358 bp); the two *C*. *albicans* samples were also PCR positive (CA1 and CA2 in Fig. 5A, the ITS amplified fragment of *C*. *albicans* was 298 bp).

**Table 6:** Tp and CV values of three randomized clinically positive samples detected by LAMP-microfluidic chip

	Sample A		Sample B		Sample C	
	Tp value*	CV/%	Tp value*	CV/%	Tp value*	CV/%
Positive quality control	17.00 ± 0.50	2.94	17.50 ± 0.50	2.86	15.67 ± 1.26	8.04
Positive amplification	24.50 ± 1.32	5.39	34.33 ± 1.26	3.67	29.67 ± 1.89	6.37

Sample A, B and C were three random samples used to evaluate the performance of the chip

^*^Tp, time of positive. Tp value represented Tp mean ± standard deviation. *CV* Coefficient of variation

Therefore, the PCR test results were consistent with the LAMP-microfluidic chip detection results, confirming that the detection accuracy of the non-culture methods, including PCR and LAMP chip method, were higher than that of the hospital standard culture method.

## Discussion

RTI during pregnancy threatens the health of both the mother and fetus, making it an urge for early and appropriate detection ahead of time. *S*. *agalactiae*, *E*. *faecalis*, *G*. *vaginalis*, *C*. *albicans* and *C*. *trachomatis* are the five main or severe pathogens in both the obstetric ward and the genital secretions of pregnant women. The classical culture, PCR and LAMP techniques can be used for the detection of these pathogens, but they are limited in routine diagnosis due to their disadvantages. The classical culture method usually takes more than 24 h to obtain the culture and even later for the detection results. LAMP approach shortens the detection time and is more tolerant of inhibitors than PCR [40], but it requires a more complicated reaction system, which usually needs at least four primers. In addition, the WHO criteria of the guide for developing point-of-care tests (POCT) includes being Affordable, Sensitive, Specific, User-friendly, Rapid, Equipment free, and Delivered to users (ASSURED) [41], therefore the integration of LAMP and microfluidic chip promotes the simultaneously visualized and rapid detection of multiple pathogens at a user-friendly manner, and this may greatly improve the efficiency and fitness of LAMP detection method, as well as its eventual application in on-site rapid screening.

There has been a surge of LAMP applications with the emergence of severe acute respiratory syndrome coronavirus 2 (SARS-CoV-2) due to the urgent practical need for virus screening [42–44]. Multi-target LAMP detection has been relatively widely used in the detection of the foodborne pathogens [33, 39, 45], but the detection of multiple pathogens in the reproductive tract during pregnancy is rather rare, largely because of the difficulty of obtaining samples from pregnant women for method validation and evaluation. Sun et al. [46] proved that high-throughput multiplex gene detection system of Sexually Transmitted Infections (STI-HMGS) was an efficient approach for the semi-quantitative detection of six important curable sexually transmitted pathogens by discriminating the length of target fragments in the products. In this study, the LAMP-microfluidic chip could simultaneously and rapidly detect and distinguish all five target pathogens in clinic samples through primer fixation and fluorescence real-time detection, and report the results within 90 min. The consistency of chip and PCR results shows that the molecular detection of clinical screening of genital tract pathogens during pregnancy is more sensitive than hospital culture method (Fig. 5).

The LAMP-microfluidic chip detection method developed in the current study went through in-tube and on-chip experiments, and the chip detection of clinical samples to further confirm its applicability. In the tube and on the chip experiments, *E*. *coli*, *Enterococcus faecium*, *Staphylococcus epidermidis*, *Streptococcus dysgalactia*, *Klebsiella pneumoniae*, *Bacteroides fragilis*, *Proteus mirabilis*, *Enterobacter cloacae*, *S*. *aureus* and *Streptococcus parahaemolyticus* did not have nonspecific amplification. Importantly, there was no cross-reaction between the five target bacteria in the chip experiment. Regarding the sensitivity, a testing with three replicates of a series of tenfold dilutions has been widely accepted, but Moehling et al. [47] proposed that estimating the limit of detection (LOD) by detecting the minimum concentration with a 95% probability (19 out of 20 were amplified) was more appropriate. Although the sensitivities of the tube and chip reaction were within 10^5^ CFU/mL, it is still worth the further improvement. As for the repeatability of the method and the stability of the chip, the CV values were less than 10%, indicating that the performance of the method and chip developed was stable. The LAMP-microfluidic chip for simultaneous detection of five targets with satisfactory specificity and sensitivity (Fig. 4), excellent clinical suitability and stability (Table 4-Table 6), and the convenient operation demonstrated its powerful potential in clinical application of preliminary screening in the simultaneous detection of multiple pathogens during pregnancy, posing great significance for the health of pregnant women and fetuses.

In the current approach for POCT, nucleic acid extraction was not integrated to achieve one-step diagnosis, otherwise it may further shorten the detection time and simplify the detection process. The breakthrough of the bottleneck depends on the upgrading of the chip and is conducive to improve the applicability of this research in POCT. At last, the optimization of basic primers and the addition of loop primers are key factors for speeding up the LAMP detection and improving the specificity and efficiency of LAMP detection, and the systematic investigation, which has been lacking, is under our current efforts [48].

## Conclusion

We have successfully developed a novel method based on LAMP-microfluidic chip for the simultaneous detection of *S*. *agalactiae*, *E*. *faecalis*, *G*. *vaginalis*, *C*. *albicans*, and *C*. *trachomatis* in female RTI. The LAMP-microfluidic chip method presented can achieve the rapid detection of these five target pathogens, and the whole process from DNA extraction to the completion of amplification took only about 90 min, with the detection sensitivities being within 10^5^ CFU/mL or copies/mL. Compared with the classic culture method, the LAMP-microfluidic chip developed greatly shortens the detection time with high specificity, efficiency and sensitivity, providing references for clinical screening and a guarantee for safe pregnancy. In addition, the easy experimental operation and reaction conditions of the LAMP-microfluidic chip can eliminate the constraints of high professionalism and be suitable to the underdeveloped medical conditions or the screening to a large population base. Further investigation of increasing clinical sample size and stability evaluation to enhance its clinical applicability for its certification is in need.

## Methods

### Materials

The standard strains and clinical isolates were used for both tube and chip experiments to establish the detection method. The source information of the standard strains of *S*. *agalactiae*, *E*. *faecalis*, *G*. *vaginalis*, *C*. *albicans* and *C*. *trachomatis* is shown in Table 7.

**Table 7:** Traceability of five standard strains

Strain	Strain traceability	Source
*Streptococcus agalactiae*	CICC 10,465	China Common Microbial Culture Collection Management Center
*Enterococcus faecalis*	CGMCC 1.10682	China Industrial Microbial Culture Collection Management Center
*Gardnerella vaginalis*	ATCC 14,018	Beina Chuanglian Biotechnology Company
*Candida albicans*	CGMCC 2.4159	China Industrial Microbial Culture Collection Management Center
*Chlamydia trachomatis* (Serotype E)	ATCC VR-348B	American Type Culture Collection

Ten clinical strains used for specificity tests, including *E*. *coli*, *Enterococcus faecium*, *Staphylococcus epidermidis*, *Streptococcus dysgalactia*, *Klebsiella pneumoniae*, *Bacteroides fragilis*, *Proteus mirabilis*, *Enterobacter cloacae*, *S*. *aureus* and *Streptococcus parahaemolyticus*, were isolated from clinical female RTI. Target strains, *S*. *agalactiae*, *E*. *faecalis*, *G*. *vaginalis*, *C*. *albicans* and *C*. *trachomatis* isolated from the hospital laboratory, were used for the positive coincidence rate measurement. HELA cells, the host cell of *C. trachomatis*, were kindly provided by Chengdu Medical College.

The clinical samples were used to evaluate the clinical applicability of the chip. A total of 103 genital tract discharge swabs were collected in 2019, samples and clinical test results were provided by the Department of laboratory medicine, Jinjiang District, the second West China hospital. All clinical samples collected were approved by the Medical Ethics Committee of West China Second Hospital, Sichuan University (2,019,083) and written informed consent was obtained from patients and their families before collection. All methods were conducted in accordance with the principles of the Declaration of Helsinki.

### Pathogen culture and quantification

The lyophilized standard strains (*S*. *agalactiae*, *E*. *faecalis*, *G*. *vaginalis* and *C*. *albicans*) and cryopreserved clinical isolates were grown on appropriate agar plate, after which single colonies were picked for rejuvenation. Particularly, *C. trachomatis* was mixed with Hela cells inoculated on 2 mL DMEM high glucose medium (GIBCO company) containing 10% fetal bovine serum and 50 μg/mL gentamicin (Macleans Biochemical Technology), and centrifuged to promote entry [49]. The 6-well plate was incubated in a 37 °C, 5% CO_2_ incubator for 48 h, and the cells were collected to obtain *C. trachomatis*. The cultured pathogen solution, except *C*. *trachomatis*, was diluted 10 times gradient with sterile phosphate buffered saline (PBS) solution, and then quantified with colony forming units (CFU).

Considering the difficulty in *C. trachomatis* quantification, the *gyrA* gene fragments were amplified by PCR and the concentration of the purified PCR products (Gel extraction kit, Omega) was determined (Thermo NanoDrop 2000). Then the copy number was calculated according to the length of the target gene fragments (http://cels.uri.edu/gsc/cndna.html), and the ten-fold gradient dilution was used as the LAMP reaction template.

### DNA extraction and PCR

DNA was extracted by using Tiangen common bacterial genome extraction kit (TIANGEN Biotech, Beijing, China) for tube experiments and Baicare Nucleic Acid Extraction Kit (Baicare Biotechnology Company, Beijing, China) for the chip experiments. The amount of 1 mL pathogen solution was used for every extraction. The extracted DNA was aliquoted and stored at -20 °C.

The fragments of the target genes were PCR amplified for the repeatability tests. All the PCR primers were listed in Additional File 4. PCR amplification system included 25 μL of PCR MIX, 19 μL of sterile water, 2 μL each of forward primer and reverse primer, and 2 μL of DNA. The PCR conditions included 94 °C for 3 min, followed by 35 cycles of 95 °C for 30 s, 56 °C for 30 s, and 72 °C for 40 s, with a final extension step at 72 °C for 7 min. The purified PCR products (Gel extraction kit, Omega) were quantified as the amplification templates mentioned above and stored for detection. The target fragments of *S. agalactiae*, *E. faecalis*, *G. vaginalis*, *C. albicans* and *C. trachomatis* are 385, 504, 358, 298 and 1334 bp.

### Primer designing and screening

The target genes were screened in the National Center for Biotechnology Information (NCBI) database. Then the primers were designed in PrimerExplorer V5 (http://primerexplorer.jp/lampv5e/index.htmL) and re-screened at NCBI Primer-Blast. The selected LAMP primer sets were synthesized by Invitrogen (Shanghai, China) with PAGE purification.

### Loop-mediated isothermal amplification

The 25 μL of the LAMP reaction mixture was composed of 12.5 μL of LAMP MIX (Baicare Biotechnology Company, Beijing, China), 2 μL of DNA, the LAMP primers with the final concentration of 1.6 μM of each FIP/BIP (0.8 μM for *G. vaginalis*), 0.2 μM of each F3/B3 and 0.4 μM of LF/LB, and sterile water. The reaction tubes were incubated in a Bio-Rad CFX96 at 64 °C for 60 min followed by 85 °C for 5 min for enzyme inactivation. Sterile water was used as the template of blank control.

### LAMP-microfluidic chip for RTI pathogens

The LAMP-Microfluidic chip of female RTI integrated LAMP reactions of positive and negative quality controls and five target pathogens. The supporting equipment, iChip-400 microfluidic fluorescent nucleic acid rapid detector, was designed and manufactured by Baicare Biotechnology Company (Beijing, China). The chip was 75.0 ± 0.5 mm in length, 28.0 ± 0.5 mm in width and 3.0 ± 0.5 mm in thickness. There were multiple reaction pools on the chip, with pools 1 to 6 for the positive quality control, *S*. *agalactiae*, *E*. *faecalis*, *G*. *vaginalis*, *C*. *albicans* and *C*. *trachomatis* in turn. In the chip prototype trial, the mixture containing the primers of corresponding pathogens, agarose and ultrapure water was added into the reaction pool of the chip after quality testing. The chips with the mixture were naturally dried before mechanically spot with corresponding reaction substrate and integrated closely with the cover. The qualified assembled chips were then sealed in a tin foil bag and stored at 4 °C till using. The mixture of 35 μL DNA sample and 35 μL LAMP MIX was injected into the chip, which was then incubated at 37 °C for 3 min followed by 65 °C for 60 min. The iChip-400 recognized the real-time fluorescence curve of the amplification, recorded the positive amplification time (time of positive, Tp). After the amplification, iChip-400 automatically output a normalized fluorescence curve, and showed whether the test result was positive or negative.

### Specificity, sensitivity, repeatability and positive coincidence rate

To evaluate the specificity of the designed LAMP primers, 10 RTI related pathogens, including *E*. *coli*, *Enterococcus faecium*, *Staphylococcus epidermidis*, *Streptococcus dysgalactia*, *Klebsiella pneumoniae*, *Bacteroides fragilis*, *Proteus mirabilis*, *Enterobacter cloacae*, *S*. *aureus* and *Streptococcus parahaemolyticus*, was amplified in parallel, melt curve step was added to analyze the LAMP products.

The DNA of five standard strains, *S*. *agalactiae*, *E*. *faecalis*, *G*. *vaginalis*, *C*. *albicans* and *C*. *trachomatis* was diluted and used as amplification templates to determine the sensitivity. The sensitivity tests were repeated three times.

The repeatability of tube experiments were evaluated by amplifying target fragments of five pathogens for 20 repetitions. For chip experiments, DNA extracted from target strains at 3 times and 30 times of the sensitivities, corresponding to weak positive and medium positive concentration, was used as templates for 10 repetitions. The Ct values for tube experiment and Tp values for the chip experiment of the amplifications were counted, and the CV (standard deviation/ mean value × 100%) values were calculated.

For the positive coincidence rate measurement, the DNA of 25 target clinical isolates, five strains for each target pathogen, was used to evaluate the positive coincidence rate of the detection method for clinical strains.

### The chip experiment of clinical sample

The swab samples of lower genital tract secretions from pregnant women with suspected reproductive tract infections were detected by microbiological testing in the hospital and LAMP-microfluidic chip testing in the laboratory. The clinical samples were placed in 1 mL normal saline to elute the pathogens and Baicare kit was used to extract DNA for LAMP-microfluidic chip experiment. Then, the detection effect of the chip on clinical samples was evaluated by the negative coincidence rate, positive coincidence rate and total coincidence rate. Negative (positive) coincidence rate = the number of samples with negative (positive) results jointly tested by LAMP and hospital culture method/total number of samples with negative (positive) results tested by hospital culture method × 100%. Total coincidence rate = the number of samples with consistent results obtained from the LAMP test and the hospital culture method test/the total number of samples tested by the hospital culture method × 100%.

In order to determine the stability of the LAMP-microfluidic chip detection of clinically positive samples, three repeated detections were performed on three random clinical samples with positive results detected by LAMP-microfluidic chip. The detection Tp values of the quality control and the targets were counted, and the CV values were calculated. For samples whose chip results were inconsistent with clinical ones, PCR was carried out for verification.

## Supplementary Information

**Additional file 1.** Detection results of clinical samples using LAMP-Microfluidic Chip.

**Additional file 2.** Original gel of PCR products in the validation experiments of clinical samples with positive chip results for *C*. *albicans*.

**Additional file 3.** Original gel of PCR products in the validation experiments of clinical samples with positive chip results for *G*. *vaginalis*.

**Additional file 4.** PCR primers of target genes.

## Data Availability

All data generated or analysed during this study are included in this published article and its supplementary information files.

## Abbreviations

RTI: Reproductive tract infection
*S*. *agalactiae*: *Streptococcus agalactiae*
*E*. *faecalis*: *Enterococcus faecalis*
*G*. *vaginalis*: *Gardnerella vaginalis*
*C*. *albicans*: *Candida albicans*
*C*. *trachomatis*: *Chlamydia trachomatis*
LAMP: Loop-mediated isothermal amplification
PCR: Polymerase chain reaction
WHO: World Health Organization
*E*. *coli*: *Escherichia coli*
*S*. *aureus*: *Staphylococcus aureus*
PBS: Phosphate buffered saline
CFU: Colony forming units
DNA: Deoxyribonucleic acid
NCBI: National Center for Biotechnology Information
Ct: Cycle threshold
CV: Coefficient of variation
Tp: Time of positive
*glk*: Glucokinase
ITS2: Internal transcribed spacer 2
POCT: Point-of-care tests
ASSURED: Affordable, Sensitive, Specific, User-friendly, Rapid, Equipment free, and Delivered
SARS-CoV-2: Severe acute respiratory syndrome coronavirus 2
LOD: Limit of detection
STI-HMGS: High-throughput multiplex gene detection system of sexually transmitted infections
