# Supplementary material for: Rapid and simultaneous detection of multiple pathogens in the lower reproductive tract during pregnancy based on loop-mediated isothermal amplification-microfluidic chip
Source: BMC Microbiol. 2022 Oct 29;22:260. doi: 10.1186/s12866-022-02657-0 (PMC9616700; doi:10.1186/s12866-022-02657-0)
Supplement: Supplementary file 1 — Additional file 1. Detection results of clinical samples using LAMP-Microfluidic Chip. [file 12866_2022_2657_MOESM1_ESM.doc]

**Additional file 1.** Detection results of clinical samples using LAMP-Microfluidic Chip

| Sample | GBS | EF | GV | CA | CT | Compliant or not * | Conc. of DNA（ng/μL） | A260/A280 |
| --- | --- | --- | --- | --- | --- | --- | --- | --- |
| 2 | − | − | − | − | − | Com | 181.2 | 1.57 |
| 3 | − | − | − | − | − | Com | 5.6 | 1.61 |
| **4** | **−** | **+** | **−** | **−** | **−** | **Com** | **124.9** | **1.68** |
| 6 | − | − | − | − | − | Com | 4 | 1.99 |
| 7 | − | − | − | − | − | Com | 12 | 1.57 |
| 8 | − | − | − | − | − | Com | 99.6 | 1.21 |
| 9 | − | − | − | − | − | Com | 136.5 | 1.71 |
| 10 | − | − | − | − | − | Com | 9.8 | 1.17 |
| 12 | − | − | − | − | − | Com | 9.9 | 1.56 |
| 13 | − | − | − | − | − | Com | 4.1 | 2.12 |
| 14 | − | − | − | − | − | Com | 5.6 | 1.85 |
| 15 | − | − | − | − | − | Com | 6.2 | 1.70 |
| 16 | − | − | − | − | − | Com | 373.4 | 1.55 |
| **17** | **−** | **−** | **+** | **−** | **−** | **Com** | **13.4** | **1.37** |
| 18 | − | − | − | − | − | Com | 6.9 | 1.59 |
| 19 | − | − | − | − | − | Com | 140 | 1.56 |
| 20 | − | − | − | − | − | Com | 12.9 | 1.51 |
| **21** | **−** | **+** | **−** | **−** | **−** | **Com** | **62.4** | **1.76** |
| 22 | − | − | − | − | − | Com | 8.9 | 1.81 |
| 23 | − | − | − | − | − | Com | 29.7 | 1.37 |
| 24 | − | − | − | − | − | Com | 63.5 | 0.90 |
| 25 | − | − | − | − | − | Com | 16.8 | 1.38 |
| 26 | − | − | − | − | − | Com | 18.5 | 1.42 |
| 27 | − | − | − | − | − | Com | 174.6 | 1.69 |
| 28 | − | − | − | − | − | Com | 44.6 | 1.32 |
| 29 | − | − | − | − | − | Com | 79.8 | 1.16 |
| 30 | − | − | − | − | − | Com | 45.1 | 1.16 |
| 31 | − | − | − | − | − | Com | 38.2 | 1.32 |
| 32 | − | − | − | − | − | Com | 9.6 | 1.46 |
| 35 | − | − | − | − | − | Com | 5.9 | 1.81 |
| 36 | − | − | − | − | − | Com | 66.1 | 1.05 |
| 37 | − | − | − | − | − | Com | 14.4 | 1.31 |
| 38 | − | − | − | − | − | Com | 97 | 0.88 |
| 39 | − | − | − | − | − | Com | 48.3 | 1.05 |
| 40 | − | − | − | − | − | Com | 22.9 | 1.48 |
| 41 | − | − | − | − | − | Com | 20.8 | 1.50 |
| 42 | − | − | − | − | − | Com | 21.3 | 1.35 |
| 44 | − | − | − | − | − | Com | 91 | 1.68 |
| 46 | − | − | − | − | − | Com | 4.4 | 1.95 |
| 47 | − | − | − | − | − | Com | 7.6 | 2.33 |
| 48 | − | − | − | − | − | Com | 8.7 | 1.78 |
| 49 | − | − | − | − | − | Com | 4.1 | 2.36 |
| 50 | − | − | − | − | − | Com | 4.4 | 1.85 |
| 51 | − | − | − | − | − | Com | 59.1 | 1.95 |
| 52 | − | − | − | − | − | Com | 4.8 | 1.82 |
| 53 | − | − | − | − | − | Com | 127.8 | 1.12 |
| 54 | − | − | − | − | − | Com | 193.7 | 1.87 |
| 57 | − | − | − | − | − | Com | 15.9 | 1.12 |
| 58 | − | − | − | − | − | Com | 10.3 | 1.41 |
| 59 | − | − | − | − | − | Com | 27.1 | 1.47 |
| 61 | − | − | − | − | − | Com | 39.8 | 1.52 |
| 65 | − | − | − | − | − | Com | 50.1 | 1.41 |
| 68 | − | − | − | − | − | Com | 30.9 | 1.39 |
| 69 | − | − | − | − | − | Com | 5.2 | 1.61 |
| 70 | − | − | − | − | − | Com | 22.5 | 1.38 |
| 71 | − | − | − | − | − | Com | 21.8 | 1.43 |
| 73 | − | − | − | − | − | Com | 5.2 | 1.88 |
| 74 | − | − | − | − | − | Com | 9.1 | 1.72 |
| 75 | − | − | − | − | − | Com | 3.9 | 2.21 |
| 76 | − | − | − | − | − | Com | 25.2 | 1.46 |
| 77 | − | − | − | − | − | Com | 17.1 | 1.44 |
| 78 | − | − | − | − | − | Com | 53.8 | 1.36 |
| 79 | − | − | − | − | − | Com | 35 | 1.33 |
| 81 | − | − | − | − | − | Com | 29.7 | 1.30 |
| 82 | − | − | − | − | − | Com | 28.8 | 1.28 |
| 84 | − | − | − | − | − | Com | 22 | 1.30 |
| 85 | − | − | − | − | − | Com | 25.4 | 1.37 |
| 86 | − | − | − | − | − | Com | 4.9 | 1.92 |
| 88 | − | − | − | − | − | Com | 20.8 | 1.59 |
| 90 | − | − | − | − | − | Com | 27.4 | 1.35 |
| 91 | − | − | − | − | − | Com | 3.9 | 2.13 |
| 92 | − | − | − | − | − | Com | 14.7 | 1.45 |
| 93 | − | − | − | − | − | Com | 19.3 | 1.51 |
| 94 | − | − | − | − | − | Com | 12.1 | 1.46 |
| 95 | − | − | − | − | − | Com | 27.7 | 1.51 |
| 96 | − | − | − | − | − | Com | 70 | 1.37 |
| 97 | − | − | − | − | − | Com | 13.6 | 1.62 |
| 98 | − | − | − | − | − | Com | 25.9 | 1.46 |
| 99 | − | − | − | − | − | Com | 27.5 | 1.43 |
| 100 | − | − | − | − | − | Com | 24.6 | 1.40 |
| 101 | − | − | − | − | − | Com | 31.7 | 1.45 |
| 102 | − | − | − | − | − | Com | 45 | 1.33 |
| 105 | − | − | − | − | − | Com | 30.5 | 1.45 |
| 106 | − | − | − | − | − | Com | 11.6 | 1.71 |
| 107 | − | − | − | − | − | Com | 46.7 | 1.46 |
| 108 | − | − | − | − | − | Com | 36.6 | 1.48 |
| 109 | − | − | − | − | − | Com | 26.4 | 1.62 |
| 112 | − | − | − | − | − | Com | 32.6 | 1.37 |
| 113 | − | − | − | − | − | Com | 24.7 | 1.4 |
| 114 | − | − | − | − | − | Com | 64.1 | 1.43 |
| 115 | − | − | − | − | − | Com | 34.3 | 1.43 |
| 116 | − | − | − | − | − | Com | 20.3 | 1.61 |
| 117 | − | − | − | − | − | Com | 32.6 | 1.45 |
| 118 | − | − | − | − | − | Com | 58.2 | 1.55 |
| 119 | − | − | − | − | − | Com | 22 | 1.55 |
| **1** | **−** | **−** | **−** | **+** | **−** | **Non-Com** | **109.8** | **1.62** |
| **5** | **−** | **−** | **+** | **−** | **−** | **Non-Com** | **119.1** | **1.73** |
| **11** | **−** | **−** | **+** | **−** | **−** | **Non-Com** | **164.5** | **1.72** |
| **33** | **−** | **−** | **+** | **−** | **−** | **Non-Com** | **111.9** | **1.73** |
| **72** | **−** | **−** | **+** | **−** | **−** | **Non-Com** | **14.3** | **1.53** |
| **80** | **−** | **−** | **−** | **+** | **−** | **Non-Com** | **20** | **1.38** |
| **103** | **−** | **−** | **+** | **−** | **−** | **Non-Com** | **10.9** | **1.54** |
| **111** | **−** | **−** | **+** | **−** | **−** | **Non-Com** | **286.3** | **1.58** |

*S. agalactiae* (GBS), *E. faecalis* (EF), *G. vaginalis* (GV), *C. albicans* (CA), *C. trachomatis* (CT). *Com, compliant; Non-Com, non-compliant.
