# Supplementary figures and images for: Rapid and simultaneous detection of multiple pathogens in the lower reproductive tract during pregnancy based on loop-mediated isothermal amplification-microfluidic chip
Source: BMC Microbiol. 2022 Oct 29;22:260. doi: 10.1186/s12866-022-02657-0 (PMC9616700; doi:10.1186/s12866-022-02657-0)

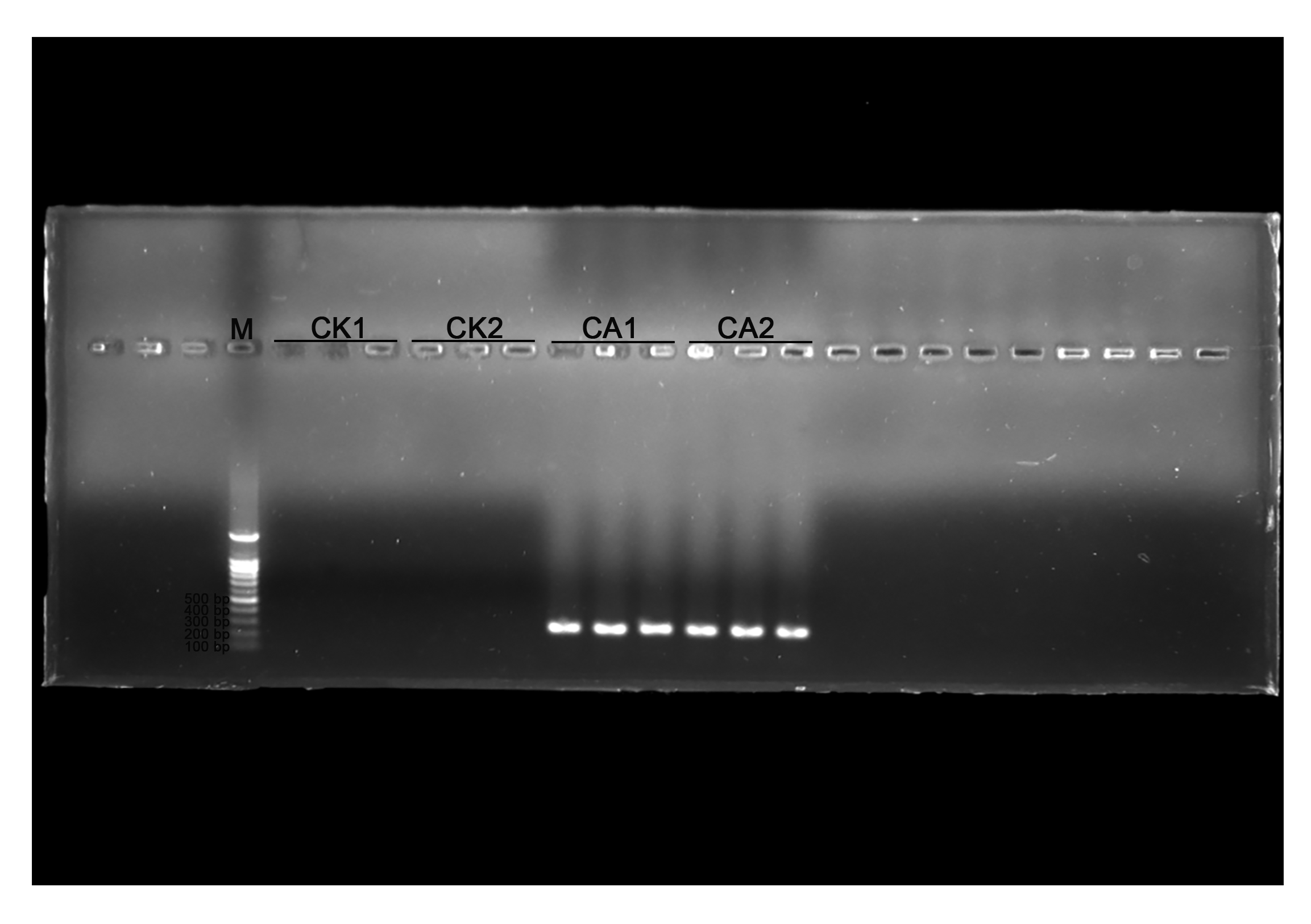

Supplement: Supplementary file 2 — Additional file 2. Original gel of PCR products in the validation experiments of clinical samples with positive chip results for C. albicans. [file 12866_2022_2657_MOESM2_ESM.tif]

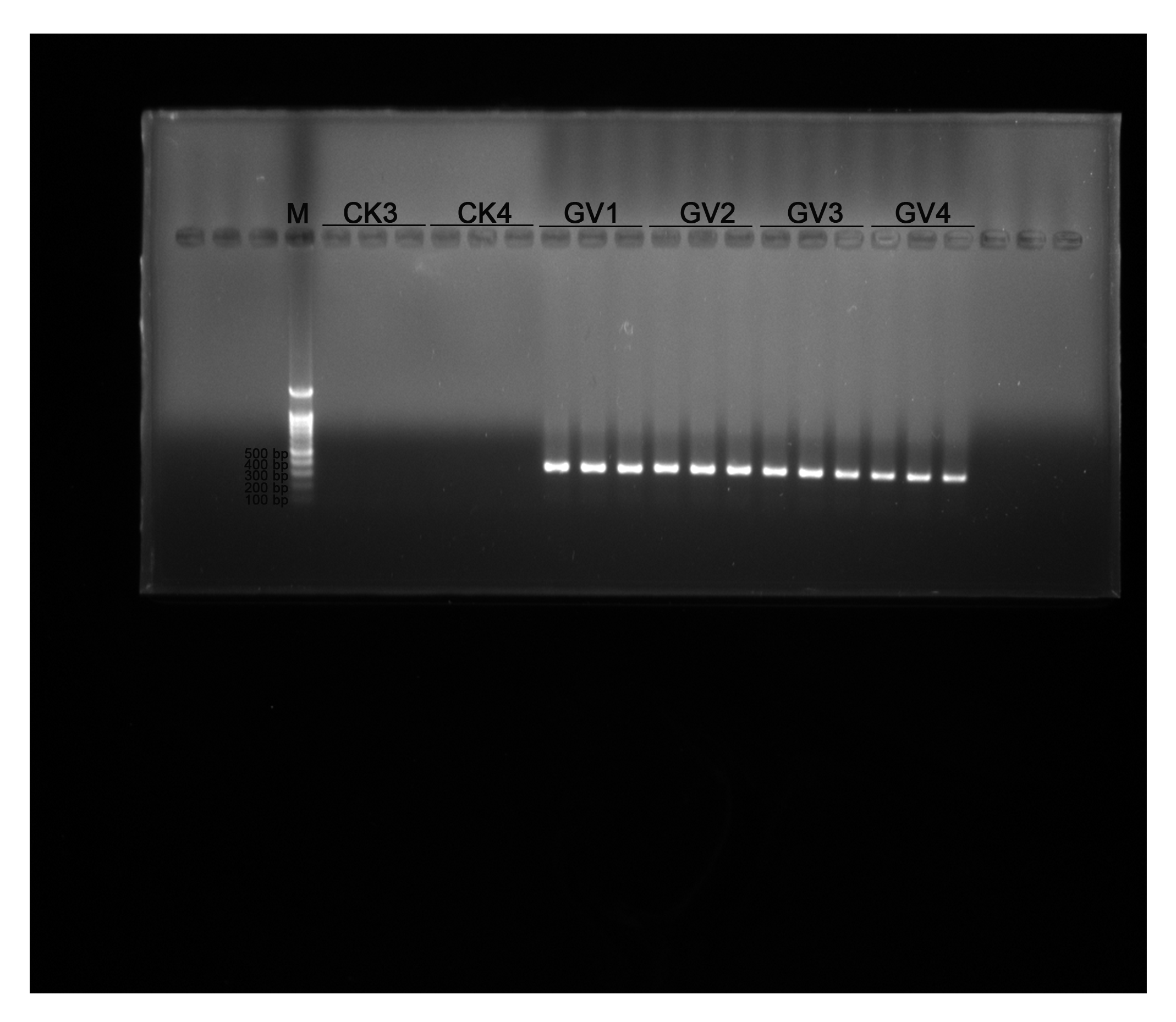

Supplement: Supplementary file 3 — Additional file 3. Original gel of PCR products in the validation experiments of clinical samples with positive chip results for G. vaginalis. [file 12866_2022_2657_MOESM3_ESM.tif]
