# Supplementary material for: Rapid and simultaneous detection of multiple pathogens in the lower reproductive tract during pregnancy based on loop-mediated isothermal amplification-microfluidic chip
Source: BMC Microbiol. 2022 Oct 29;22:260. doi: 10.1186/s12866-022-02657-0 (PMC9616700; doi:10.1186/s12866-022-02657-0)
Supplement: Supplementary file 4 — Additional file 4. PCR primers of target genes. [file 12866_2022_2657_MOESM4_ESM.doc]

**Additional file 4.** PCR primers of target genes

| Strain | Primer sequence |
| --- | --- |
| *Streptococcus agalactiae* | F: TGGTATCTTGACGCTTGAGG |
|  | R: CGATAACACCTCCACCTACTCC |
| *Enterococcus faecalis* | F: GCGATTATTTCTGTTGAGGG |
|  | R: CGGATAATGACGCCTTTG |
| *Gardnerella vaginalis* | F: CGTCCGCTGAATGACTAAG |
|  | R: GGAATCAGCCTTACAGCCTAC |
| *Candida albicans* | F: CCGCCAGAGGTCTAAACTTAC |
|  | R: GCCTTACCACTACCGTCTTTC |
| *Chlamydia trachomatis* | F: GCACTTTCTTGTATGGACGAAG |
|  | R: CCCAGATTGAACACTTGCTG |
